# Shift in Patterns of Care and Survival Outcomes of Hepatocellular Carcinoma in a Canadian Provincial Cancer Program

**DOI:** 10.7759/cureus.104989

**Published:** 2026-03-10

**Authors:** Nikunj Patil, Amelia Baker, Christie Nashed, Gaby Rizk, Miray Eskandar, Hang Yu, David Peretz, Mariam Shenouda, Alessandra Cassano-Bailey

**Affiliations:** 1 Radiation Oncology, CancerCare Manitoba, Winnipeg, CAN; 2 Radiation Oncology, University of Manitoba, Winnipeg, CAN; 3 Max Rady College of Medicine, University of Manitoba, Winnipeg, CAN; 4 School of Medical Sciences, University of Manchester, Manchester, GBR; 5 Radiology, University of Manitoba, Winnipeg, CAN; 6 Hepatology, University of Manitoba, Winnipeg, CAN; 7 Internal Medicine, University of Toronto, Toronto, CAN

**Keywords:** canada, hepatocellular carcinoma (hcc), retrospective studies, survival, transarterial chemoembolization (tace)

## Abstract

Purpose

Hepatocellular carcinoma (HCC) is the most common and highly morbid primary liver malignancy. In this retrospective study, we describe the clinical characteristics of patients with HCC, identify factors determining the type of delivered treatment, and evaluate the overall survival (OS).

Materials and methods

Demographic and treatment details were collected for HCC patients diagnosed in Manitoba between January 2011 and December 2021. The cohort was divided according to the treatment that was delivered: curative, non-curative, and supportive care-only groups.

Results

A total of 800 patients were identified. Complete staging information was not available for all patients. The mean age was 67 years, and men represented 72.5% of the cohort. One hundred ninety-five (24.4%) patients received curative treatment, 188 (23.5%) received non-curative treatment, and 417 (52.1%) received supportive care only. Alcoholic cirrhosis was the most common etiology of chronic liver disease (20.3%). Patients were less likely to receive curative treatment if they had portal hypertension or multifocal disease. Patients with ascites or distant metastasis were more likely to receive supportive care only. The one- and five-year OS rates for the full cohort were 50% and 23%, respectively. Between 2011 and 2021, the prevalence of non-curative therapies has increased. Trans-arterial chemoembolization (TACE) was the most common non-curative treatment with one- and three-year OS rates of 88% and 41%, respectively.

Conclusion

The OS of patients with HCC in Manitoba improved between 2011 and 2021 and was temporally associated with increased utilization of non-curative local therapies, including TACE. However, given the retrospective nature of the study, these findings demonstrate association rather than causal effect.

## Introduction

Hepatocellular carcinoma (HCC) is the most common primary liver malignancy, with a global annual incidence of 905,677 and an annual mortality of 830,000 [1,2]. In Canada, HCC is the second fastest growing cancer in incidence [3]. In countries with a high Human Development Index (HDI), the incidence of HCC has been increasing, possibly caused by an increasing prevalence of alcoholic cirrhosis and metabolic disorders, such as obesity and type 2 diabetes mellitus [4,5]. 

Over the past two decades, treatment options for HCC have significantly expanded [6]. In response, various professional organizations, such as the American Association for the Study of Liver Diseases (AASLD) [7] and the Barcelona Clinic Liver Cancer (BCLC) [8], have issued their recommendations for the management of HCC for varying stages of disease. 

Treatment allocation in HCC is primarily stage-driven, guided by the BCLC staging system, which integrates tumor burden, liver function, and performance status to inform prognosis and therapeutic strategy. Clinical outcomes are strongly dependent on treatment type and intent [9]. For example, patients with BCLC 0 and A disease treated with hepatic resection or radiofrequency ablation (RFA) have a five-year overall survival (OS) rate of 40%-70% [10]. Local non-curative treatment options include trans-arterial chemoembolization (TACE) and stereotactic body radiation therapy (SBRT), which are associated with three-year OS rates of 35% and 44%, respectively [11].

In Manitoba, the implementation of locoregional therapies for HCC was relatively delayed, with the introduction of TACE in 2015 and SBRT in 2016. However, provincial data describing longitudinal treatment patterns and associated survival trends are lacking. This retrospective study therefore aims to characterize the clinical features of patients diagnosed with HCC in MB, identify factors influencing treatment decisions, and evaluate the association of non-curative local therapies on survival. 

## Materials and methods

Study design and population

This retrospective study included Manitoba residents diagnosed with HCC between January 2011 and December 2021. Ethics approval was obtained from the Health Research Ethics Board of the University of Manitoba. Eligible patients, according to the International Classification of Diseases, 10th revision, Code C22.0, were identified through the Manitoba Cancer Registry (MCR), which is a population-based registry containing information regarding all cancer diagnoses since 1956 [12,13]. Patients with radiologically diagnosed or histologically confirmed HCC, including mixed histology, were included in the study. Patients with liver metastases or other types of primary liver malignancy were excluded.

Data collection

Patient demographics, tumor characteristics, and treatment details were collected from electronic medical records and clinical documentation from the Department of Hepatology at the University of Manitoba. Tumor characteristics, such as size and focality, were collected from radiological reports (ultrasound, CT, or MRI). Complete staging information (BCLC staging and Child-Pugh score) was not available for all patients.

Patients were categorized into three groups based on treatment intent: (1) curative (hepatic resection, liver transplantation, or ablation), (2) non-curative (TACE, SBRT, or systemic therapies), and (3) supportive care alone. 

Statistical analysis

Descriptive statistics were used to summarize patient characteristics and treatment types. Group comparisons were conducted using analysis of variance (ANOVA), χ^2^ test, Fisher’s exact test, and Kruskal-Wallis tests. Survival was measured as the time between the date of diagnosis and the date of death or the last date of contact. Survival was calculated using the Kaplan-Meier method, with June 30, 2024, designated as the study endpoint. Two-year survival estimates were calculated by diagnosis year and plotted. Temporal trends were assessed using natural cubic splines in a Cox regression model. Changes in treatment distribution over time were evaluated using a χ^2^ test. All analyses were run using R version 4.4.1 (R Foundation for Statistical Computing, Vienna, Austria) and the rms package.

## Results

Patient characteristics

A total of 800 patients were included. The mean age at diagnosis was 67 years, and 72.5% of the patients were men (Table 1). Cirrhosis was present in 77.6% of the cohort, with alcohol use being the most common etiological factor (20.3%). One hundred ninety-five (24.4%) patients received curative treatment; 188 (23.5%) received non-curative treatment, the most common being TACE (114 patients; 14.3%); while 417 (52.1%) patients received supportive care alone.

**Table 1:** Characteristics of the study cohort (N = 800) *One patient underwent palliative surgery. NASH: non-alcoholic steatohepatitis, HCC: hepatocellular carcinoma.

Cohort characteristics	n (%)
Mean age at diagnosis (years)	67
Gender	
Male	580 (72.5%)
Female	220 (27.5%)
Etiology	
Alcohol	162 (20.3%)
Hepatitis B	50 (6.3%)
Hepatitis C	98 (12.3%)
Other (hemochromatosis, NASH, autoimmune)	76 (9.5%)
Mixed etiology	135 (16.9%)
Unknown	279 (34.9%)
Cirrhosis	621 (77.6%)
HCC severity	
Metastasis	92 (11.5%)
Treatment	
Curative treatment	
Liver transplant	6 (0.8%)
Resection/surgery	90 (11.3%)
Radiofrequency ablation (RFA)	99 (12.4%)
Non-curative treatment*	
Trans-arterial chemoembolization (TACE)	114 (14.3%)
Stereotactic body radiotherapy (SBRT)	19 (2.4%)
Systemic therapy	54 (6.8%)
Supportive care	417 (52.1%)

Factors influencing treatment allocation

**Figure 1:**
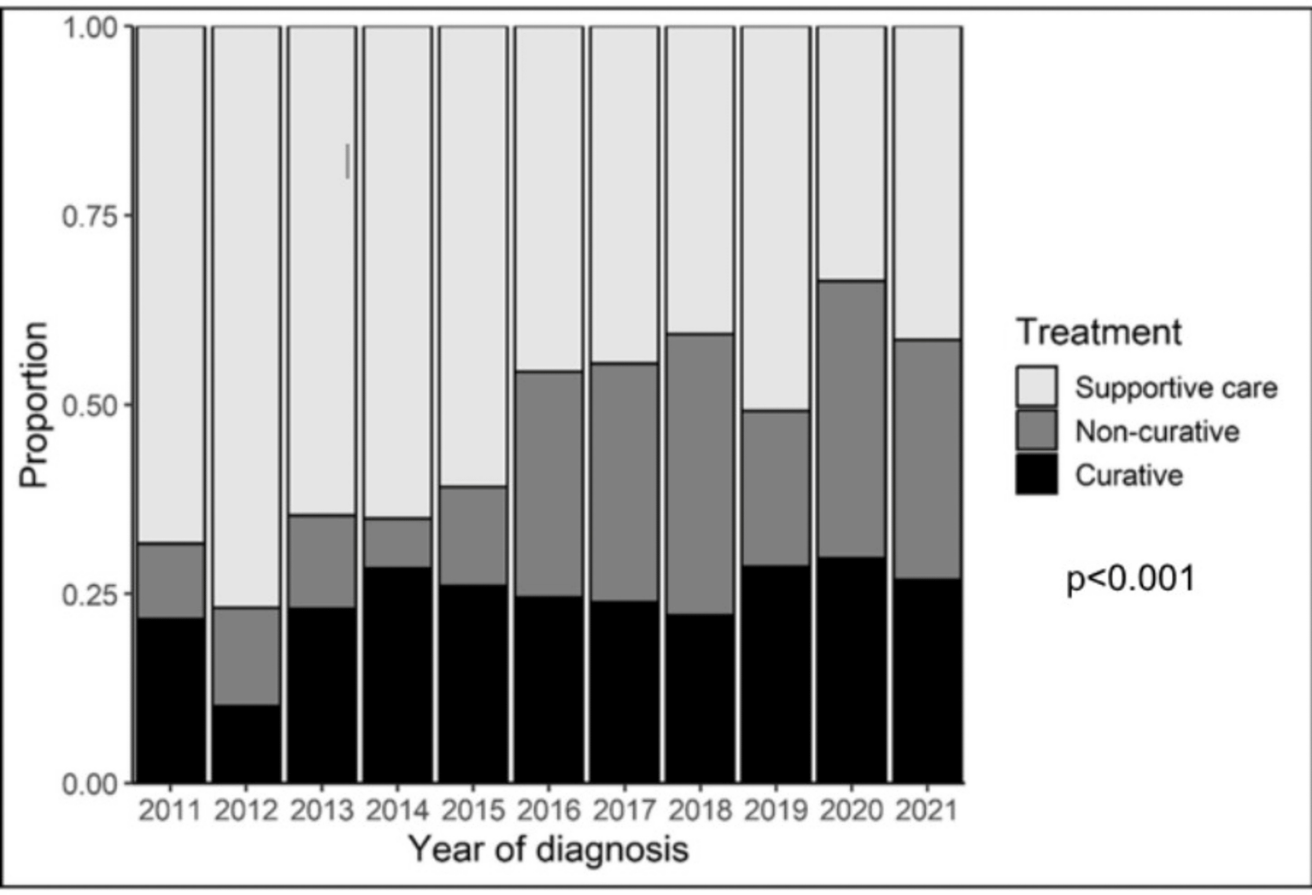
Proportion receiving treatment by year of diagnosis

Univariate analyses identified several clinical variables associated with treatment modality choice (Table 2). Patients with unifocal disease and absence of portal vein thrombosis (PVT) were more likely to receive curative treatment. Those who received supportive care alone were typically older, had ascites, had larger tumors, or metastatic disease. Patients with no portal hypertension were more likely to receive a curative-intent therapy. The distribution of treatment types significantly changed over the study period (p < 0.001), with an increased uptake of non-curative therapies (Figure 1).

**Table 2:** Univariate analyses of the association of different variables with the given treatment

Variable	Full cohort	Curative	Non-curative	Supportive care	p-value	Significance of pairwise comparison
Mean age at diagnosis, years (SD)	67 (10.7)	64.3 (9.7)	66.3 (9.3)	68.5 (11.5)	<0.001	Curative vs supportive care; non-curative vs supportive care
Number of foci						
Single	354 (44.3%)	138 (70.8%)	73 (38.8%)	143 (34.3%)	<0.001	Curative vs non-curative; curative vs no supportive care
Multiple	412 (51.5%)	52 (26.7%)	112 (59.6%)	248 (67.4%)		
Unknown	34 (4.3%)	5 (2.6%)	3 (1.6%)	26 (6.2%)		
Portal vein thrombosis						
Yes	161 (20.1%)	10 (5.1%)	40 (21.3%)	111 (26.6%)	<0.001	Curative vs non-curative; Curative vs supportive care
No	600 (75%)	178 (91.3%)	141 (75%)	281 (67.4%)		
Unknown	39 (4.9%)	7 (3.6%)	7 (3.7%)	25 (6%)		
Portal hypertension						
Yes	437 (54.6%)	90 (46.2%)	101 (53.7%)	246 (59%)	0.003	Curative vs supportive care
No	327 (40.9%)	99 (50.8%)	80 (42.65%)	148 (35.5%)		
Unknown	36 (4.5%)	6 (3.1%)	23 (5.5%)	23 (5.5%)		
Ascites						
Yes	267 (33.4%)	29 (14.9%)	32 (17%)	206 (49.4%)	<0.001	Curative vs supportive care; non-curative vs supportive care
No	495 (61.9%)	160 (82.1%)	149 (79.3%)	186 (44.6%)		
Unknown	38 (4.8%)	6 (3.1%)	7 (3.7%)	25 (6 %)		
Cirrhosis						
Yes	621 (77.6%)	153 (78.5%)	151 (80.3%)	317 (76%)	0.96	
No	146 (18.3%)	36 (18.5%)	34 (18.1%)	76(18.2%)		
Unknown	33 (4.1%)	6 (3.1%)	3 (1.6%)	24 (5.8%)		
Metastasis						
Yes	92 (11.5%)	1 (0.5%)	12 (6.4%)	278 (66.7%)	<0.001	Curative vs supportive care; non-curative vs supportive care; curative vs supportive care
No	611 (76.4%)	179 (91.8%)	154 (81.9%)	278 (66.7%)		
Unknown	97 (12.1%)	15 (7.7%)	22 (11.7%)	60 (14.4%)		
Tumor size						
Median (cm)	4.6	2.6	4.6	6.4	<0.001	Curative vs supportive care; non-curative vs supportive care; curative vs supportive care
Unknown (n)	51	3	4	44		

Survival outcomes

**Figure 2:**
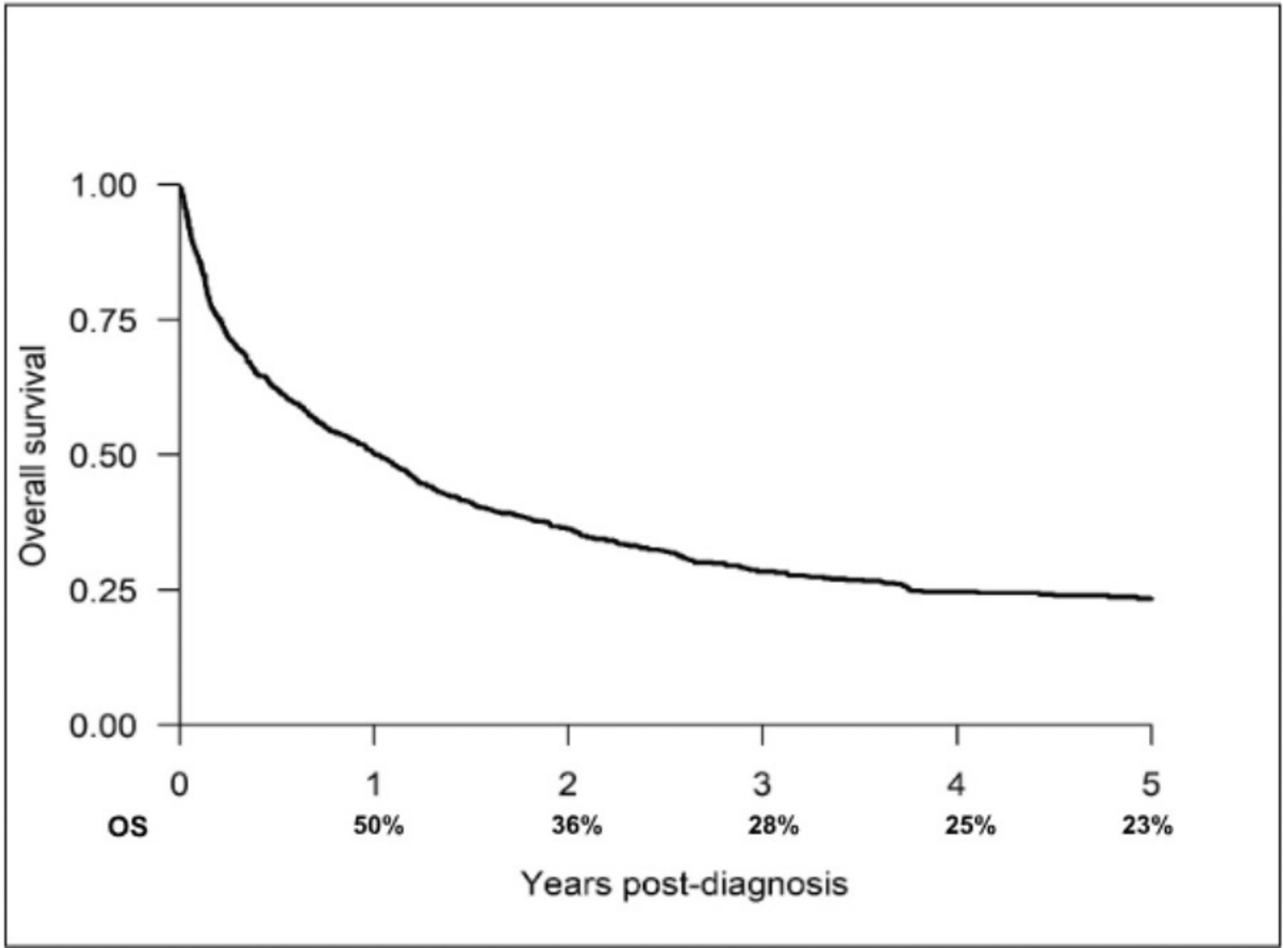
Overall survival rates of the whole cohort

The OS rates for the entire cohort at one, two, three, four, and five years were 50%, 36%, 28%, 25%, and 23%, respectively (Figure 2).

**Figure 3:**
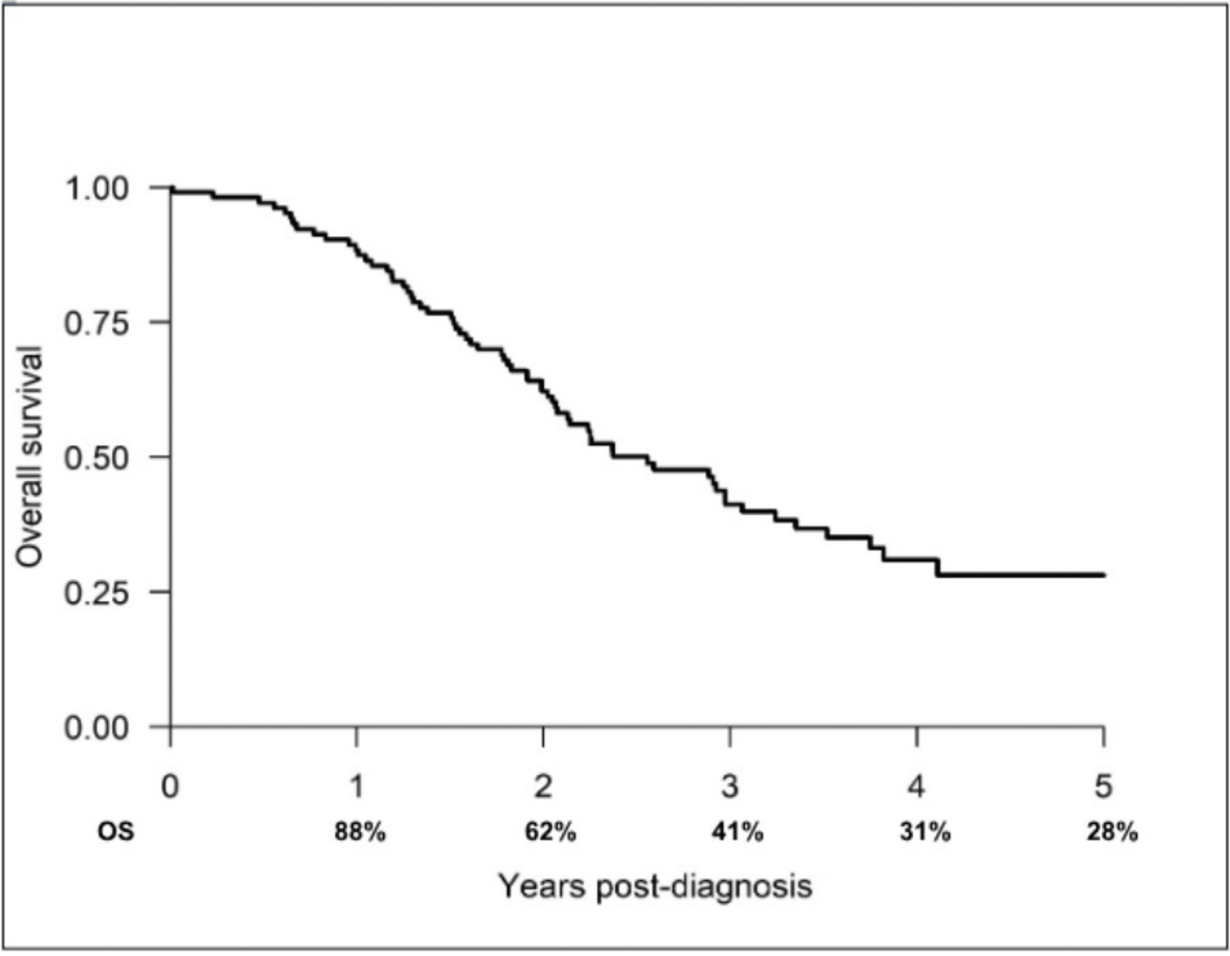
Overall survival rates of patients who received TACE TACE: trans-arterial chemoembolization.

Among the 114 patients treated with TACE between 2015 and 2021, the one-, two-, three-, four-, and five-year OS rates were 88%, 62%, 41%, 31% and 28%, respectively (Figure 3).

## Discussion

This retrospective study provided insights into the clinical characteristics, treatment patterns, and survival outcomes of patients who were diagnosed with HCC in Manitoba between January 2011 and December 2021.

Within our cohort (800 patients), alcohol use was the most common (20.3%) single cause of cirrhosis, consistent with findings reported by several other studies [14-16]. Viral hepatitis was the second most common etiology (18.6%). Notably, in 2019, Manitoba had a higher hepatitis C rate (55.6 per 100,000) than the Canadian average (33.9 per 100,000) [17]. This trend may be related to immigration patterns. In Canada, immigrants account for at least 20% of all cases of HCV [18] and have a two- to threefold higher incidence of HCC compared to the general population [19-21]. The etiology of cirrhosis was undetermined for 34.9% of the patients. 

Although 77.6% patients had cirrhosis, its presence did not influence treatment decisions. Patients with multifocal disease or PVT were less likely to receive curative treatment, which is expected, as those patients belong to BCLC stage A or higher. 

According to the European Association for the Study of the Liver (EASL) guidelines [22], prognosis is not only impacted by disease burden but also by performance status and liver function (defined by Child-Pugh score). Unifocal disease, absence of PVT, and absence of portal hypertension were associated with receiving curative therapy, while ascites, a marker of liver decompensation and advanced BCLC stage, was associated with receiving supportive care alone.

There has been a notable improvement in survival outcomes over time. In a previous report [23], we measured the one- and five-year OS for patients diagnosed between 2011 and 2015 at 41% and 14%, respectively. This has now improved to 50% and 23% for the entire 2011 and 2021 cohorts. The five-year OS in Manitoba is now comparable with the Canadian average of 22% [24]. This improvement is likely attributed to the use of non-curative therapies, in particular TACE, that became available in 2015. Over time, there has been an increase in the proportion of patients receiving non-curative therapies, except for those diagnosed in 2019 (Figure 2). Different potential reasons could be postulated to explain this observation, such as the lockdown during the COVID-19 pandemic.

TACE was the most frequently administered non-curative therapy (14.3%). The one- and five-year OS for patients treated with TACE were 88% and 28%, respectively. These results are consistent with prior studies by Burrel et al. [25] and Llovet et al. [26], who reported a one-year survival of TACE-treated patients of 88.2% and 82%, respectively. We believe the improved OS rate in the entire cohort, compared to the cohort diagnosed between 2011 and 2015 [23], is driven by the survival benefit gained from the non-curative local therapies especially TACE, which was the most common non-curative treatment modality.

Strengths of this study include detailed clinical characterization of key factors such as tumor size, the presence of portal hypertension, PVT, multifocality, ascites, and metastases, which all influence treatment decisions. The findings suggest the clinical benefit of non-curative therapies, particularly TACE, in improving OS.

A key limitation of this study is the incomplete availability of staging data, including BCLC stage and Child-Pugh score. These factors are central to both treatment allocation and prognosis. Their absence precludes multivariable analysis and may confound observed associations between treatment patterns and survival, including temporal improvement. In addition, data were analyzed according to the first treatment delivered; however, subsequent or crossover treatments may significantly influence survival, and failure to account for them may confound interpretation. This precluded the use of time-dependent treatment modeling. Furthermore, this study reflects a single-center experience, which may limit generalizability. Nonetheless, the results are consistent with published literature and underscore the importance of expanding access to locoregional therapies for HCC. 

## Conclusions

The OS of patients with HCC in Manitoba improved between 2011 and 2021 and was temporally associated with increased utilization of non-curative local therapies, including TACE. However, given the retrospective nature of the study, these findings demonstrate association rather than causal effect. 
